# Uncovering the Fecal Bacterial Communities of Sympatric Sika Deer (*Cervus nippon*) and Wapiti (*Cervus canadensis*)

**DOI:** 10.3390/ani12182468

**Published:** 2022-09-18

**Authors:** Jiakuo Yan, Xiaoyang Wu, Xibao Wang, Yongquan Shang, Honghai Zhang

**Affiliations:** College of Life Science, Qufu Normal University, Qufu 273165, China

**Keywords:** sika deer (*Cervus nippon*), wapiti (*Cervus canadensis*), fecal microbiota, alpha diversity, beta diversity

## Abstract

**Simple Summary:**

There are many microbial communities in the digestive tracts of animals, and the complex gut microbiome constitutes an intricate ecosystem and intestinal microbial community which has co-adapted with its host. The intestinal microecology plays an important role in the host’s maintenance of normal physical activities, such as substance metabolism, energy transmission, signal transduction, and the immune system. This study used high-throughput sequencing technology to sequence the fecal microbiota of sika deer (*Cervus nippon*) and wapiti (*Cervus canadensis*) in order to explore the composition of, and the similarity between, the fecal microbiota structures of sika deer and wapiti in the similar living environment. The species composition, relative abundance of fecal microbiota, alpha diversity, and differences in beta diversity were analyzed. The maintenance of the composition of the gut microbiota and a balanced intestinal environment through the diet plays a key role in maintaining the host’s health. The results demonstrate that the fecal microbiota of sika deer and wapiti share a similar fecal microbiota structure, but there was some evidence showing that the gut microbiota of these two animals exhibit a clear divergence at the species level.

**Abstract:**

Microbial symbiotic associations may be beneficial, neutral, or harmful to the host. Symbionts exploit the host space and nutrition or use hosts as carriers to spread to other environments. In order to investigate the fecal bacterial communities of wild sika deer (*Cervus nippon*) and wapiti (*Cervus canadensis*), this study aimed to sequence and explore the composition of, and similarity between, the fecal microbiota of sika deer and wapiti using high-throughput sequencing. The composition and relative abundance of fecal microbiota, alpha diversity, and differences in beta diversity between the two species were analyzed. We found that no pathogenic bacteria were present in large quantities in the hosts. The dominant bacterial phyla found in the two deer species were similar and included Firmicutes, Bacteroidetes, Proteobacteria, and Spirochaetes. Moreover, the deer also shared similar dominant genera, including the *Rikenellaceae RC9* gut group, *Ruminococcaceae_UCG-010*, *Ruminococcaceae_UCG-005*, and Bacteroides. These results demonstrate that the sika deer and wapiti share a similar fecal microbiotal structure, probably due to their common diet and living environment, but there was some evidence of a difference at the species level. These analyses provide new insights into the health status of deer populations outside protected environments and offer a scientific framework for monitoring the health conditions of sika deer and wapiti.

## 1. Introduction

**Figure 1:**
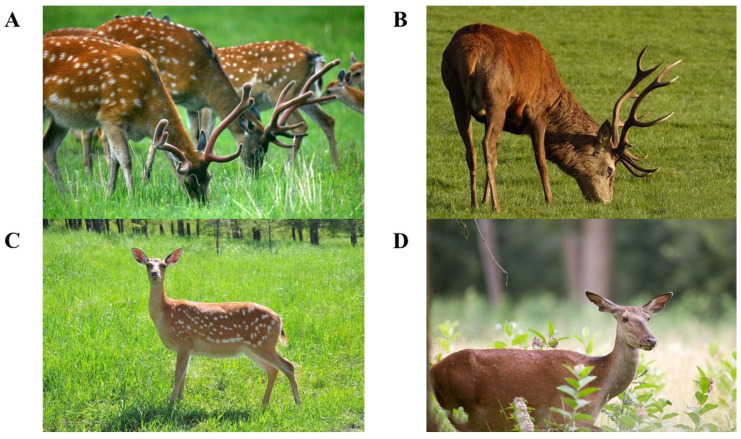
(**A**) Male sika deer. (**B**) Male wapiti. (**C**) Female sika deer. (**D**) Female wapiti.

Although the earliest appearance of the Cervidae family can be traced back to the late Oligocene period [1], the cold-resistant Cervidae appeared during the late Pliocene period owing to a climate-cooling event. The fossils of ancient *Cervus* from the late Pliocene and Pleistocene periods portray very similar characteristics to those of modern-day sika deer. The modern-day wapiti, however, are believed to have originated from the ancient wapiti during the early Pleistocene period [2]. The sika deer (*Cervus nippon*) have spread across East Asia, from central China in the west to Japan and Korea in the east, and from the extreme Eastern tip of Russia in the north to China and Vietnam in the south [3]. The female and male Sika deer and wapiti demonstrate numerous differences in terms of morphology and physiology; for example, the male deer have antlers, the bony appendages shown in Figure 1.

Sika deer mainly inhabit woodlands and forests with dense understories. They are active in the forest during summer and migrate to lower valleys during winter. Their main diet consists of grass and fruits [4]. They are crepuscular and forage for food either individually or in small herds, with the dominant males possessing harems [5]. Owing to habitat fragmentation, weak habitat connectivity, and the reduction in the forest area, the distribution and size of the population of sika deer have decreased sharply. Three subspecies of sika deer found in China have been listed among the national class I key protected animals. They are listed as a least-concern species in the International Union for Conservation of Nature (IUCN) Red List of Threatened Species [6].

Researchers believe that the wapiti (*Cervus canadensis*) are related to the sika deer. The primitive wapiti evolved from the primitive sika deer population when it spread from the Middle East to Europe and northern Africa. However, the primitive wapiti returned to China’s mainland through the Tianshan Mountains during the middle of the Pleistocene period [7]. During this process of geographical expansion, the body size of the wapiti gradually increased, and their antler types diversified. Generally, wapiti inhabit open coniferous forests, deciduous forests, and mixed deciduous–coniferous forests. However, they have also been found in upland moors, open mountainous areas, pastures, and natural grasslands [8]. In the woodlands, their primary sources of food include shrubs and tree shoots, while in other habitats, their diets consist of grasses and sedges. However, they are also known to consume fruits and seeds during autumn [9]. The wapiti living in mountainous regions spend their summers in the alpine meadows and winters in valleys, while those inhabiting other flat terrain occupy wooded hillsides in the summer and open grasslands in the winter. The wapiti found west of Central Asia are known to occupy the woody bushes along the riverbanks in the desert [10].

Herbivorous mammals face several challenges, as they rely on plants as their primary source of food. Up to 60% of plants are composed of indigestible cell wall substances, such as cellulose, hemicellulose, and lignin, which are not easily digested by the endogenous digestive enzymes of animals [11]. Additionally, some secondary metabolites produced by plants reduce the digestibility and alter the homeostasis, thereby affecting digestion in herbivores [12]. To aid in the consumption of plants, herbivores form symbiotic relationships with microbial communities. The main functions of these microorganisms include the digestion and fermentation of food and aiding in the degradation of high-molecular-weight polymers, such as cellulose, into short-chain fatty acids that are easily absorbed by the host [13]. Studies have shown that the evolving symbiotic relationships between herbivores and intestinal microorganisms have made a significant contribution to the digestion of fiber by these animals [14]. These observations indicate that the digestion of plants by herbivores mainly depends on the microorganisms present in their intestines [15].

The Cervidae family has a strong ability to digest crude fiber feed. However, there is a lack of systematic and original research on the structure of the fecal microbiota of deer. Therefore, in this study, we explored the differences in the composition of the gut microbiota of sika deer and wapiti living in the same environment and consuming a common diet and studied the symbiotic relationship between the host and its gut microbiota. The results of this study can provide a theoretical basis for deer preservation.

## 2. Materials and Methods

A total of 14 fresh fecal samples used in this study were collected from the Shanghai Wildlife Park in China. The deer were dependent on *Sasa nipponica*, a dwarf bamboo, throughout the year, particularly in winter, when it accounted for as much as 77.7% of the diet. It accounted for 33.1% and 45.6% in spring and summer, respectively, and this decreased to 12.2% in autumn. All the graminoid categories accounted for large proportions of the diet (66–96.7%), while dicotyledonous plants accounted for little (3–8%), except in autumn, when they accounted for 31%. Fecal samples were collected from healthy adult sika deer (*Cervus nippon*) and wapiti (*Cervus canadensis*) after confirming that no antibiotics were ingested in the last three months. When collecting the fecal samples, the sex and sampling cage number of the animals were recorded in detail. Sterile collection equipment was used for the sampling, and the samples were placed in sterile sampling bags and quickly frozen in a portable refrigerator. Finally, the samples were transferred to an ultra-low-temperature refrigerator and stored at −80 °C to ensure their freshness and minimize the chance of contamination. The sampling and experimental procedures were performed in compliance with internationally recognized standards and were approved by the Biomedical Ethics Committee of the Qufu Normal University.

### 2.1. DNA Extraction and Sequencing

Microbial DNA was extracted from the feces of the two deer species using the QIAamp DNA stool mini kit (Qiagen, Hilden, Germany). The integrity and purity of the DNA samples were examined using agarose gel electrophoresis, and the DNA concentration was quantified using Qubit 2.0 (Invitrogen, Carlsbad, CA, USA). Diluted genomic DNA was used as the template. Phusion^®^ High-Fidelity PCR Master Mix with GC Buffer and a high-efficiency, high-fidelity enzyme were used to ensure a high amplification efficiency and accuracy. The DNA library was prepared using the TruSeq^®^ DNA PCR-Free Sample Preparation Kit (Illumina, San Diego, CA, USA), and the constructed library was quantified using Qubit, verified using qPCR, and then subjected to sequencing on Illumina’s MiSeq system. The raw reads were submitted to the NCBI BioProject database (accession number: PRJNA827351).

### 2.2. Quality Control of the Raw Sequences

The raw data obtained from each sample were separated according to the barcode sequence and the PCR primer sequence. FLASH (V1.2.7, http://ccb.jhu.edu/software/FLASH/, accessed on 3 January 2021) was used to splice the reads from each sample [16], and the raw tags obtained via splicing were subjected to strict filtering in order to obtain high-quality tags (clean tags) [17]. The quality control process conducted in QIIME (V1.7.0, http://qiime.org/scripts/split_libraries_fastq.html, accessed on 12 February 2021) consisted of the following operations: (1) the raw tags were truncated to the specified length (the default length value was 3) at the site of the first low-quality base with a continuous low-quality value (the default threshold value was not larger than 19); and (2) this tag data set was further filtered to remove the tags for which the continuous high-quality base length was less than 75% of the tag length. The tag sequences were aligned to the sequences in the GOLD Database (http://drive5.com/uchime/uchime_download.html, accessed on 14 April 2021) using the UCHIME algorithm to detect the chimeric sequences. Lastly, the chimeric sequences were removed to obtain the final effective sequence data [18,19].

### 2.3. OTU Clustering and Species Classification Analysis

The UPARSE software (Uparse v7.0.1001, http://drive5.com/uparse/, accessed on 11 May 2021) was used to cluster all the effective tags from all the samples. By default, sequences were clustered into operational taxonomic units (OTUs) if they showed a 97% identity. The sequences with the highest frequency in the OTUs were selected as representative sequences of the OTUs based on the algorithm of the software [20]. Next, representative sequences of the OTUs were annotated. A species annotation analysis (the threshold value was in the range of 0.8~1) was carried out using the mothur software (mothur v1.31.2: https://www.mothur.org/, accessed on 22 September 2021). The SILVA database (SILVA 132, http://www.arb-silva.de/, accessed on 5 November 2021), comprising SSU rRNA, was used to obtain the composition of the microbial communities in the sample at each taxonomic level: kingdom, phylum, class, order, family, genus, and species [21]. The MUSCLE software (Version 3.8.31, http://www.drive5.com/muscle/, accessed on 18 December 2021) was used to perform fast multiple sequence alignments so as to determine the phylogenetic relationships between all the OTU sequences (muscle -in seqs.fa -out seqs.afa, muscle -in seq.afa -clw -out seq.clw, muscle -maketree -in seqs.afa -out seqs.phy) [22]. Finally, the data from all the samples were homogenized for subsequent alpha and beta diversity analyses.

### 2.4. Complexity Analysis

Alpha diversity was used to explore the diversity of the bacterial communities within each sample. In addition, the cumulative frequency box plot of each species, species diversity index curves, and a series of statistical analyses were used to evaluate the species richness and diversity of the microbial communities [23]. Alpha diversity is usually measured using untrimmed, i.e., non-rarefied data. The species abundance in each sample was analyzed using the QIIME software by calculating the population richness index and community diversity index (observed species, Chao1, Shannon, Simpson, ACE, goods_coverage, PD_whole_tree). Using the relative proportions of the known OTUs in the 16S rDNA sequences, the expected alpha indices for extracting *N* (*N* was less than the total number of reads) were calculated. A dilution curve was generated by plotting the expected value of the alpha index against a set of *N* values (usually a set of arithmetic sequences less than the total number of sequences). Moreover, the rank abundance curve, species accumulation curve, and alpha diversity index were analyzed using the R software. The rarefaction curve is a commonly used curve that describes the rationality of the amount of sequencing data and the diversity of samples within a group. OTU rank abundance curves showed the relative species abundance of the samples and served as a reflection of the species richness and evenness [24]. The wider the span of the rank abundance curve on the horizontal axis is, the greater the species richness of the sample is, and the smoother the curve is, the more uniform the species composition of the sample is.

### 2.5. Comparative Analysis

Beta diversity refers to the between-sample diversity, and its level reflects the difference in the species composition of the microbial community between samples. The QIIME software was used to calculate the UniFrac distance and construct a sample phylogenetic tree. To visually characterize the relationships between samples, a principal component analysis (PCA) graph was plotted using the ADE4 and ggplot2 packages of R. Subsequently, a principal co-ordinates analysis (PCoA) graph was plotted using the WGCNA, stats, and ggplot2 packages of R. The unweighted UniFrac distance was calculated using the phylogenetic relationships between the OTUs, which consider only the presence or absence of species [25,26], while the weighted UniFrac distance was calculated by combining the richness of the OTUs with the phylogenetic relationships. Weighted UniFrac distances focused on the abundant species, whereas the unweighted UniFrac distances focused on the rare species. The dissimilarity coefficient, indicating the dissimilarity between samples, was calculated based on the weighted UniFrac and unweighted UniFrac distances. The smaller the value is, the lower the diversity between the two samples is. The R software was used to analyze the difference in the beta diversity index between groups and to perform parametric and non-parametric tests. Linear discriminant analysis (LDA) effect size (LEfSe) analyses were performed using the LEfSe tool (Version 1.0.8, http://huttenhower.sph.harvard.edu/lefse/, accessed on 6 January 2022), with the default filter value for the LDA score set at 4. Significant differences between species were examined using the t-test and plotted using the R software. To explore the ecological interactions between the fecal microbiota, the microbiome analysis tool MEGAN (Version 3.5, http://ab.inf.uni-tuebingen.de/software/megan/, accessed on 8 February 2022) was used to create microbial co-occurrence network plots. A network graph consists of nodes and edges. Nodes represent bacterial classifications, and the node size represents the strength of the association. Edges represent statistically significant associations and are indicated by red for positive correlations and green for negative correlations. For co-occurrence to be demonstrated, the nodes A and B must attain the minimum probability. We applied Kendall’s Tau-b correlation coefficient method to generate co-occurrence and co-exclusion graphs. The correlation was calculated using the formula: (C − D) / (C + D), where C represents the number of concordant pairs and D represents the number of discordant pairs. The positive and negative values of the Tau correlation coefficient represent co-occurrence and co-exclusion, respectively. In this study, the overall network significance threshold was set at a *p*-value of 0.05, and the edge threshold was set at 70% to control the false positive rate. Many parameters were used to measure and monitor the microbial network. For the analysis, the minimum detection threshold for a taxon to be regarded as “present” in the sample was set at 1%.

## 3. Results

### 3.1. Sequence Data Processing

Raw paired-end (PE) reads were obtained using the Illumina HiSeq sequencing platform. The lean tags obtained by filtering for quality were subjected to subsequent analysis after the removal of chimeric sequences. Statistical analyses were carried out at each step of the data processing, and the results are shown in Table 1. Abbreviations: CE.F stands for female wapiti, CE.M stands for male wapiti, CN.F stands for female sika deer, CN.M stands for male sika deer.

### 3.2. OTU Clustering and Species Annotation

To explore the diversity of the species composition of the samples, the OTUs obtained by clustering the effective tags were subjected to species annotation. First, the OTU clustering and annotation results of each sample were statistically analyzed (Figure S1). Subsequently, a phylogenetic tree was generated. The taxonomic tree generated using GraPhlAn showed the kingdom at the center, with the phylum, class, order, family, and genus branching outwards [27]. The phylogenetic analysis showed that Firmicutes, Bacteroidetes, and Proteobacteria were the most abundant phyla (Figure S2). To analyze the species classification tree, the top 10 genera with the highest relative abundance were chosen and the results were displayed using self-developed drawing tools (Figure S3) [28].

### 3.3. Distribution of Fecal Microbiota

**Figure 2:**
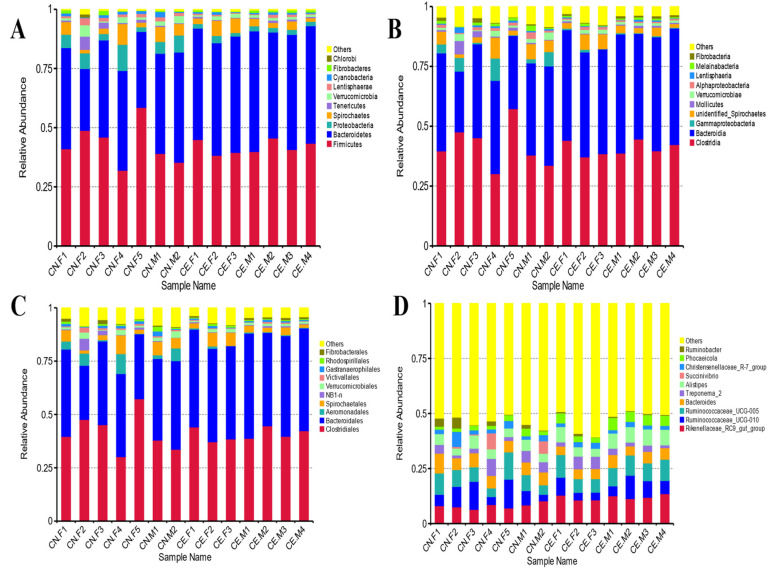
Bar plot of relative abundance. (**A**) Taxonomic level of phylum. (**B**) Taxonomic level of class. (**C**) Taxonomic level of order. (**D**) Taxonomic level of genus. Each bar represents the top ten bacterial species ranked by the relative abundance in each individual sample.

**Figure 3:**
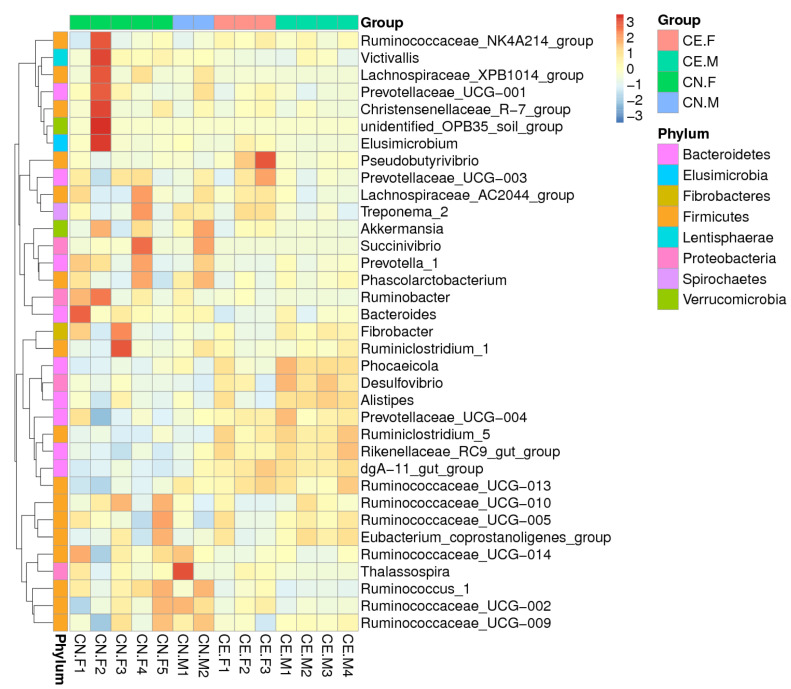
The heatmap of the clustering for species abundance. The information regarding the samples and species annotation is demonstrated along X-axis and Y-axis, respectively. The clustering tree was generated based on the relative abundance of the genera representing the top 35 most abundant bacteria at the species level. The relative values in the heatmap depicted by colors, after normalization, indicate the aggregation degree or content of bacterial species among samples at the genus level. UPMGA clustering trees.

**Figure 4:**
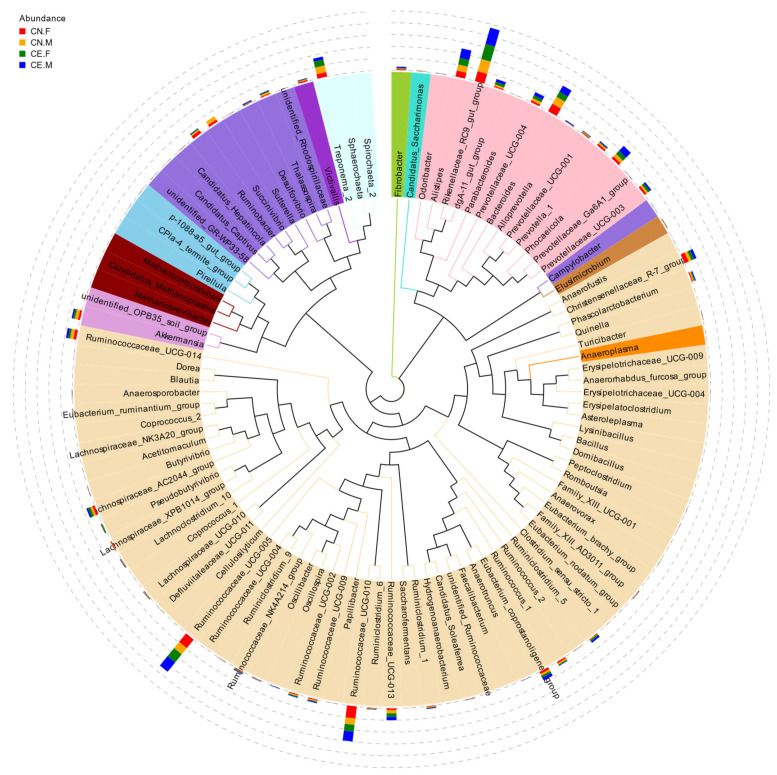
Phylogenetic relationships between species at the genus level. An evolutionary tree was established using the relative abundances at the genus level and the top 100 operational taxonomic units (OTUs). The colors represent the phyla to which the OTUs belong.

Based on the results of the species annotation, the 10 species with the highest relative abundance at the phylum, class, order, and genus taxonomic levels were selected, and a bar plot was generated to visualize the relative levels of abundance of these species. As shown in the proportional relative abundance bar chart, Firmicutes, Bacteroidetes, and Proteobacteria were the most dominant phyla of bacteria (Figure 2). Based on the species annotation results and the relative abundance of each sample at the genus level, we clustered the genera that represented the top 35 most abundant bacteria at the species level and generated a heat map to depict the results (Figure 3). To further study the changes in the specific intestinal bacterial communities, we analyzed the phylogenetic relationships between representative species at the genus level using multiple sequence alignments and selected representative sequences from the top 100 genera. As shown in Figure 4, the three genera with the highest relative abundance in both groups were *Rikenellaceae_RC9*, *Ruminococcaceae_UCG-005*, and *Alistipes*.

### 3.4. Alpha Diversity

**Figure 5:**
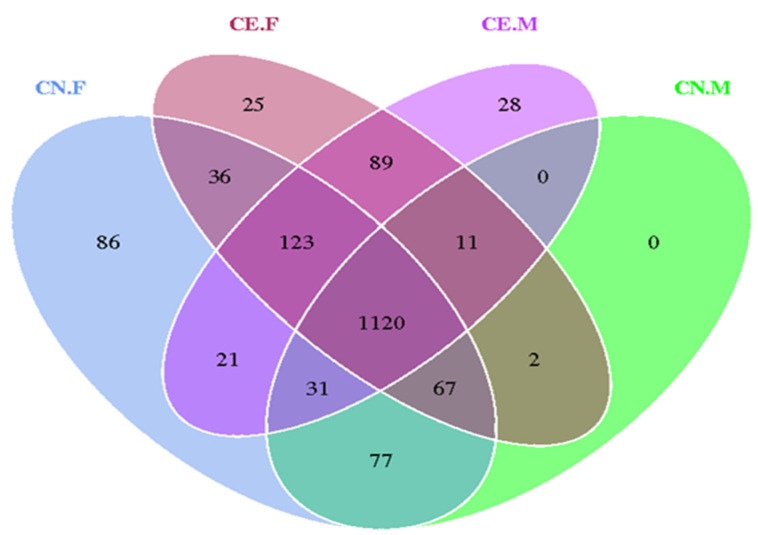
Venn graph of the OTU clustering. Venn graph shows the common and unique OTUs among the different groups. The overlapping regions indicate the number of shared OTUs.

**Figure 6:**
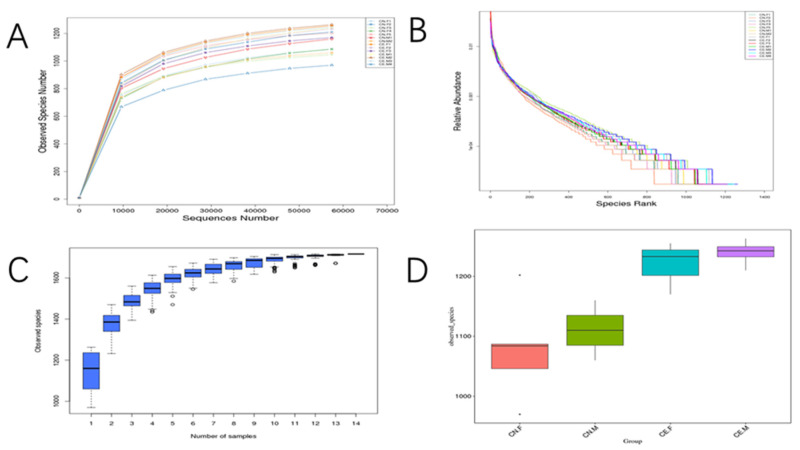
(**A**) The rarefaction curves for the species abundance of the samples. The horizontal axis represents the sequence number, and the vertical axis represents the observed species number. Each curve in the figure represents a sample, presented in a different color. (**B**) Rank abundance curves of the bacterial OTUs derived from each sample. Different samples are represented by curves of different colors. The abscissa is the species rank, and the ordinate is the corresponding relative abundance. (**C**) Species accumulation boxplot. The abscissa represents the sample size and the ordinate represents the number of OTUs after sampling. The results reflect the rate of new OTUs under continuous sampling. (**D**) Box plots of observed species.

The indices of the alpha diversity analysis, namely, the observed species, Shannon, Chao1, ACE, goods_coverage, and Simpson indices, were calculated for each sample using the Wilcoxon rank sum test (Table 2). Based on the results of the OTU cluster analysis and our research requirements, the common and unique OTUs among the different groups were analyzed using a Venn graph. The overlapping areas among the four groups represented 1120 OTUs, which were common to the four groups, out of the total number of 1716 OTUs (Figure 5). Moreover, it also showed that the inter-species differences were greater than the intra-species differences. As shown in Figure 6A, the rarefaction curve of each sample tended to saturate, indicating that the microbiome diversity of all the samples studied was captured in our research. As shown in Figure 6B, the curve spanned wide in the horizontal direction, indicating that the fecal microbiota composition of the samples was rich. The relatively high species richness showed that our results were rational and reasonable. The species accumulation box plot in Figure 6C shows that the cumulative species richness reached critical values, indicating that the sample size was adequate. Furthermore, the box plots of the alpha diversity indices shown in Figure 6D illustrate the discrepancies in the gut bacterial communities between sample groups (t-test, Wilcoxon, and Tukey tests).

### 3.5. Beta Diversity

**Figure 7:**
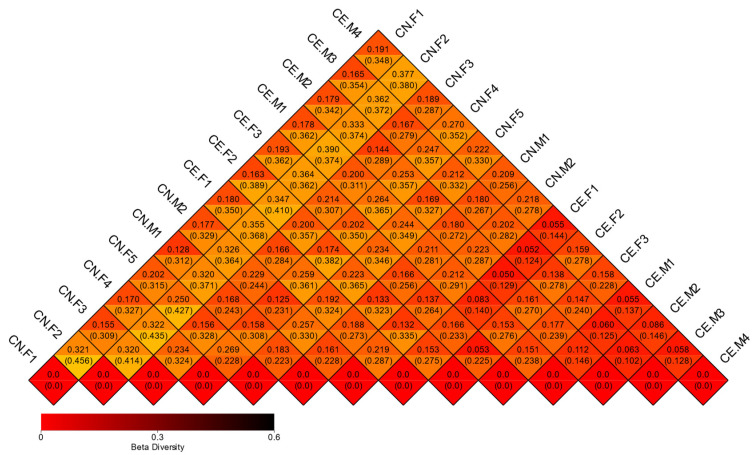
Heatmap of beta diversity. The numbers in the grids are the dissimilarity coefficients of the samples. The diversity of the bacterial species is proportional to the dissimilarity coefficient. The two numbers in the same grid represent weighted and unweighted Unifrac distances, respectively.

A beta diversity analysis was used to compare the fecal microbiota components of each sample. Weighted UniFrac and unweighted UniFrac distances were computed based on the OTU richness of the samples, and the diversity of the gut microflora among different samples was analyzed [29]. A heat map plotted based on the unweighted UniFrac and weighted UniFrac distances between samples revealed no significant differences between the fecal microbiota of female and male deer (Figure 7). The heatmap was constructed based on strain-level differences. Moreover, considering the most important elements and the species abundance information, PCA was used to further study the similarities in the fecal microbiota composition between samples (Figure S4). Furthermore, we performed a beta diversity analysis and generated a three-dimensional graph, which depicted the differences between samples to the greatest extent. Subsequently, PCoA was performed using a combination of unweighted UniFrac distances and the main coordinates. The closer the distance between samples was, the higher their similarity was. PCoA also showed that the differences in the composition of the gut microbiota were more significant between species than within species (Figure S5). Dendrograms were constructed using unweighted pair-group method using arithmetic mean (UPGMA) (Figure S6) The PCoA and PCA plots were constructed based on strain-level differences. Furthermore, a cluster analysis was conducted to determine the similarities between the gut microbial communities in the samples. A clustering tree diagram was constructed at the phylum classification level, using the unweighted pair group method with the arithmetic mean. The results revealed two main clusters in the clustering tree, with little variation between the samples (Figure 6C). In addition, the LEfSe analysis detected the differentially abundant species between groups (Figure S7). In this study, LEfSe was used to identify the unique taxa of the two groups. The results indicated that the difference in the diversity of the fecal microbiota between the two groups was not obvious (Figure 6D).

**Figure 8:**
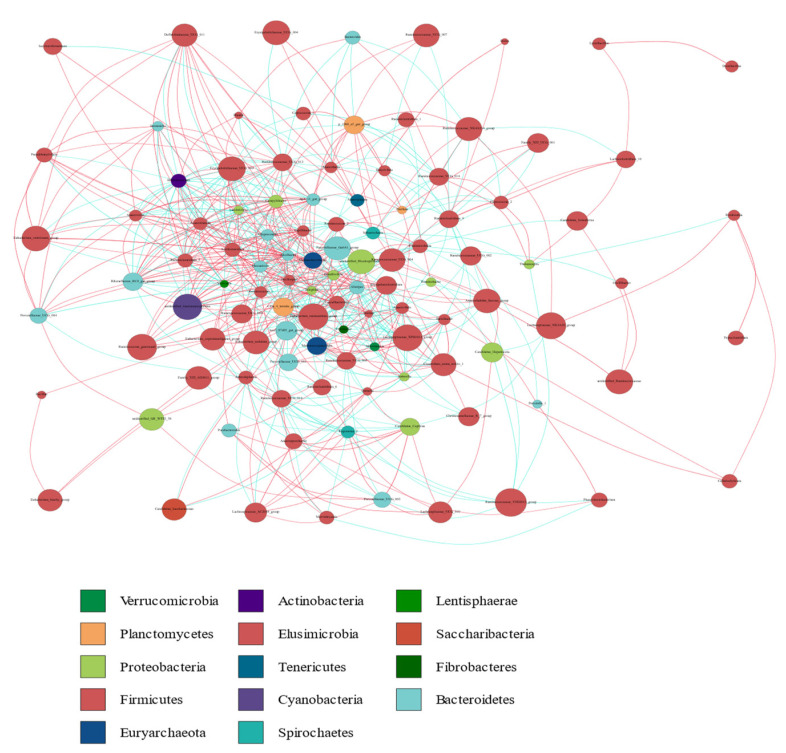
Microbial co-occurrence networks analysis. Each node represents a genus. Each edge indicates the sign of the association (green = positive (co-occurrences), red = negative (co-exclusions)). The thickness of the nodes represents the level of association between taxa.

The co-occurrence network diagram provided a new perspective for the study of the community structure and functions of the complex microbial environments. Since the co-occurrence relationships between microorganisms in different environments are different, we can study the impact of different environmental factors on microbial adaptability and identify the dominant species and closely interacting groups in an environment using the species co-occurrence network diagram. These dominant species and groups often play unique and important roles in maintaining the stability of the microbial community structure and its function in the environment. Therefore, we calculated the correlation index (Spearman correlation coefficient (SCC) or Pearson correlation coefficient (PCC)) of all the samples and presented the obtained correlation coefficients in a table. The correlation coefficients obtained were sorted using a cutoff value of 0.6, and a map was generated by combining these data with the species abundance data. Furthermore, a network analysis revealed the co-occurrence and co-exclusion relationships between the dominant bacterial genera belonging to 14 dominant bacterial phyla. The most common phyla found on the nodes were Firmicutes, Bacteroidetes, and Proteobacteria (Figure 8). This result was consistent with our aforementioned findings.

## 4. Discussion

This study showed that 16S rDNA sequencing can help us to identify uncultured bacteria and that this method can be used for the phylogenetic classification and typing of unknown bacteria. Some researchers have studied the composition of the microbial flora in the intestines of animals; however, such research materials are not easy to obtain, and their costs are often associated with the slaughtering of the specific animal. In particular, in studies involving large wild animals, these in vivo and autopsy experiments are highly unlikely to be conducted, owing to the scarcity of the animals. From the perspective of animal conservation, fecal samples are ideal non-invasive research materials. Therefore, fecal samples were selected for the study of the gut microbiota structure of the wapiti and sika deer. The experimental results obtained represent the general situation of the fecal microbiota of these animals.

Diverse intestinal microorganisms play numerous beneficial roles in the host. They promote the overall development of the host, aid in the fulfillment of the metabolic needs of the host, and regulate the immunity and reproductive behaviors of the host [30]. Various factors, such as the living environment, age, heredity, and diet, affect the fecal microbiotal composition of the host [31,32]. The interaction between the fecal microbiota and host has received increasing attention in recent years. In this study, we performed high-throughput sequencing of the highly variable region of the 16S rRNA gene in the gut microbiomes of wapiti and sika deer and found that Firmicutes and Bacteroidetes were the most abundant bacteria in the intestines of the two species, accounting for approximately 75% of the total bacteria. Firmicutes are also the dominant bacteria in the intestines of black bears, mice, and humans. Since they primarily hydrolyze carbohydrates, Firmicutes play an important role in host nutrient absorption and metabolism. The predominant bacteria in the gut microbiota of both species also included Proteobacteria, Spirochaetes, and Verrucomicrobia. At the genus level, the abundance of the *Rikenellaceae_RC9_gut_group* was high in the fecal samples, and similar findings were observed in the intestinal bacteria of other ruminants. Overall, at the phylum level, both sika deer and wapiti showed a relatively stable microbial community structure and diverse fecal microbiota. These dominant bacterial species are closely associated with nutrient metabolism, playing a major role in the digestion of various carbohydrates and improving the degradation of cellulose in the animal intestine. However, the mechanisms underlying this relationship require further study.

Researchers have analyzed the fecal microbiotal composition of mammals and found that their living environments and diet structures are the primary factors affecting the diversity of their gut microbiota [33,34,35]. In this study, in both male and female deer, we found that Firmicutes and Bacteroidetes were the most abundant bacteria at the phylum level, and Clostridiales, belonging to Firmicutes, and Bacteroidales, belonging to Bacteroidetes, had the highest abundance at the order level. The abundance ratio of these two phyla and orders amounted to approximately 75% in each of the fecal samples of both male and female deer, indicating that there is no significant difference in the core microflora structure and species abundance between the male and female deer groups. Analysis of the beta diversity and the differences in the microbial community structure in the fecal samples of male and female deer revealed no significant differences in these parameters between both groups, but the corresponding PCA plots and principal coordinate analysis (PCoA) plots showed that the gut microbiota of these two animals exhibit a clear divergence at the species level. Studies have shown that sex is an important factor in determining the diversity of the intestinal microbial community in mammals [36,37,38]. In the present study, although the species diversity was higher in the male sample group than in the female sample group, it did not reach a significant level. We speculate that this may be due to the convergent evolution of the intestinal microorganisms of the male and female deer living under the same conditions for prolonged periods of time. Previous works have discovered strong relationships both between environmental and dietary effects and the gut microbiome function and between the consumption of specific plant metabolites and fecal metabolite profiles. It seems that, in the deer, it is more important to have a shifting/transient microbiome than one that is more conserved. This likely helps the deer to adapt to seasonal dietary changes and, vice versa, seasonal diet changes likely alter and sometimes even homogenize their microbiomes.

Our results showed that Spirochetes were the dominant bacteria in the core flora of the two deer species. Research has revealed that the bacteria belonging to the genus *Treponema* of *Spirochetes* interact with cellulolytic bacteria and aid in cellulose degradation [39]. They effectively degrade the complex polysaccharides in plant cell walls into short-chain fatty acids, such as butyrate, propionate, and acetate, thereby providing energy for the host and the fecal microbiota. Treponema is an important and beneficial bacterium that suppresses the growth of maleficent bacteria, such as *Salmonella* and *Escherichia coli* [40]. It is possible that the high disease resistance characteristics of these species are associated with their unique gut microbial composition.

Proteobacteria is another major phylum in the intestines of wapiti and sika deer. This phylum comprises a variety of pathogenic Gram-negative bacteria, including *Escherichia* spp., *Campylobacter* spp., *Salmonella* spp., and *Pseudomonas* spp. [41]. These bacteria contain *lipopolysaccharide* (LPS, endotoxin) in their outer membranes, and this LPS induces a strong immune response in the host, thereby providing an early warning signal for infection by a Gram-negative pathogen.

## 5. Conclusions

In conclusion, our results demonstrate that the fecal microbiota of sika deer and wapiti share a similar structure. This may be due to the migration of sika deer and wapiti to new common environments and their adaptation to the changes in food sources and environment. In addition to the similarity in the anatomical structure of their digestive tracts and physiological metabolism, the structure and functions of their gut microbiota also tend to be similar. Therefore, the composition and diversity of the fecal microbiota are directly affected by changes in the natural environment and food, sources which influence the adaptive changes in the digestive and immune health of the deer. We must ensure that edible plants are supplied and that scientific supervision is carried out to ensure their good health. Our analyses provide new insights into the health status of deer populations in ex situ conservation sites and offer a scientific framework for monitoring the health conditions of sika deer and wapiti.

## Figures and Tables

**Table 1:** Data pre-processing and quality control.

Sample Name	Species	Sex	Raw PE	Raw Tags	Effective Tags	Base (nt)	Q20 (%)
CE.F1	Wapiti	Female	215,245	141,222	134,475	60,715,588	97.58
CE.F2	Wapiti	Female	312,835	198,233	187,510	84,818,640	97.52
CE.F3	Wapiti	Female	166,116	90,359	84,310	38,127,557	97.19
CE.M1	Wapiti	Male	405,347	259,034	243,872	110,341,845	97.53
CE.M2	Wapiti	Male	259,462	161,632	152,476	68,796,462	97.5
CE.M3	Wapiti	Male	372,031	231,191	216,808	98,053,905	97.53
CE.M4	Wapiti	Male	253,677	164,855	156,897	70,878,325	97.56
CN.F1	Sika	Female	262,154	169,901	158,383	71,622,503	97.6
CN.F2	Sika	Female	268,349	157,103	149,495	67,308,187	97.43
CN.F3	Sika	Female	182,780	91,628	85,071	38,336,278	97.14
CN.F4	Sika	Female	223,971	128,275	116,926	52,878,708	97.28
CN.F5	Sika	Female	127,220	66,761	62,767	28,159,699	97.12
CN.M1	Sika	Male	133,959	69,757	64,502	29,144,342	97.15
CN.M2	Sika	Male	162,926	89,577	82,270	37,163,066	97.2

**Table 2:** Alpha diversity of the gut microbiota in fecal samples.

Sample	Species	Sex	Observed_Species	Shannon	Simpson	Chao1	ACE	Goods_Coverage
CN.F1	Wapiti	Female	1084	7.974	0.991	1148.024	1156.249	0.998
CN.F2	Wapiti	Female	970	7.703	0.99	1044.882	1059.369	0.998
CN.F3	Wapiti	Female	1046	8.227	0.993	1100.301	1103.797	0.998
CN.F4	Wapiti	Male	1087	7.675	0.986	1190.5	1195.605	0.997
CN.F5	Wapiti	Male	1202	8.526	0.994	1251.5	1249.636	0.998
CN.M1	Wapiti	Male	1160	8.172	0.992	1294.384	1287.833	0.997
CN.M2	Wapiti	Male	1060	7.686	0.986	1113.509	1118.624	0.998
CE.F1	Sika	Female	1255	8.313	0.993	1325.522	1334.977	0.998
CE.F2	Sika	Female	1233	8.168	0.992	1365.026	1340.944	0.997
CE.F3	Sika	Female	1170	8.053	0.99	1232.046	1236.775	0.998
CE.M1	Sika	Female	1240	8.201	0.993	1390.976	1369.852	0.997
CE.M2	Sika	Female	1263	8.408	0.993	1322.049	1335.561	0.998
CE.M3	Sika	Male	1245	8.328	0.993	1319.773	1321.592	0.998
CE.M4	Sika	Male	1210	8.27	0.993	1331.454	1298.882	0.997

## Data Availability

The datasets presented in this study can be found in online repositories. The accession number(s) can be found on NCBI (accession: PRJNA827351).

## Supplementary Materials

The following supporting information can be downloaded at: https://www.mdpi.com/article/10.3390/ani12182468/s1, Figure S1: Statistical analysis of OTUs clustering and annotation for each sample; Figure S2: Hierarchical tree of the taxa composition at each taxonomic level was constructed by GraPhlAn; Figure S3: The species classification tree statistics; Figure S4: Principal component analysis (PCA) plot; Figure S5: Unweighted UniFrac-Principal coordinate analysis (PCoA); Figure S6: Dendrograms were constructed using unweighted pair-group method using arithmetic mean (UPGMA); Figure S7: Cladograms indicating the polygenetic distribution of bacterial lineages between CE.F and CN.F by linear discriminant analysis (LDA) effect size (LEfSe). Coloring principles: Species with no significant difference were uniformly colored yellow, the species of Biomarker were colored following the different group, the red node was the bacteria group that played an important role in the red group, and the green node was indicated to play an important role in the green group bacterial taxa.
