# Supplementary material for: Uncovering the Fecal Bacterial Communities of Sympatric Sika Deer (Cervus nippon) and Wapiti (Cervus canadensis)
Source: Animals (Basel). 2022 Sep 18;12(18):2468. doi: 10.3390/ani12182468 (PMC9495088; doi:10.3390/ani12182468)
Supplement: Supplementary file 1 [file animals-12-02468-s001.zip › animals-1845514-supplementary.pdf]

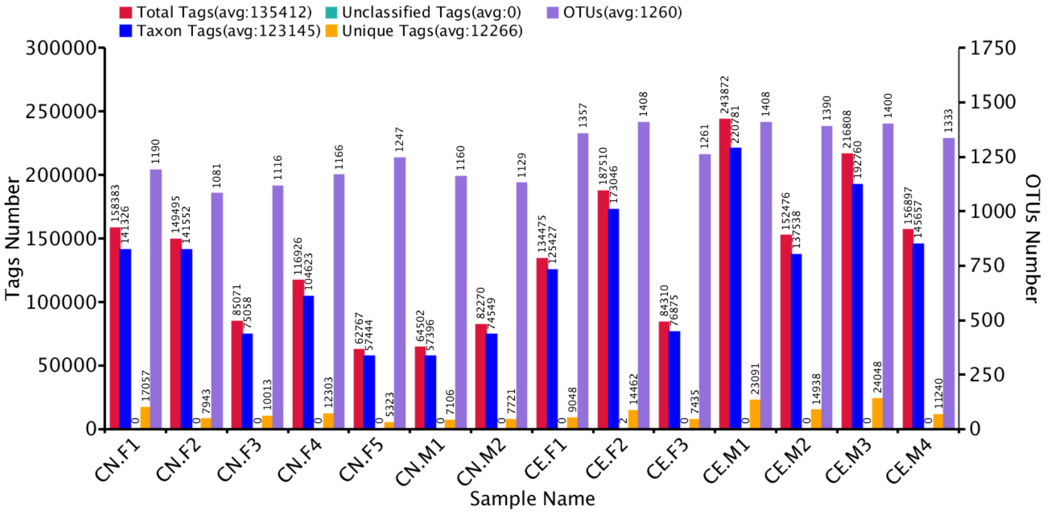

Figure S1 | Statistical analysis of OTUs clustering and annotation for each sample.

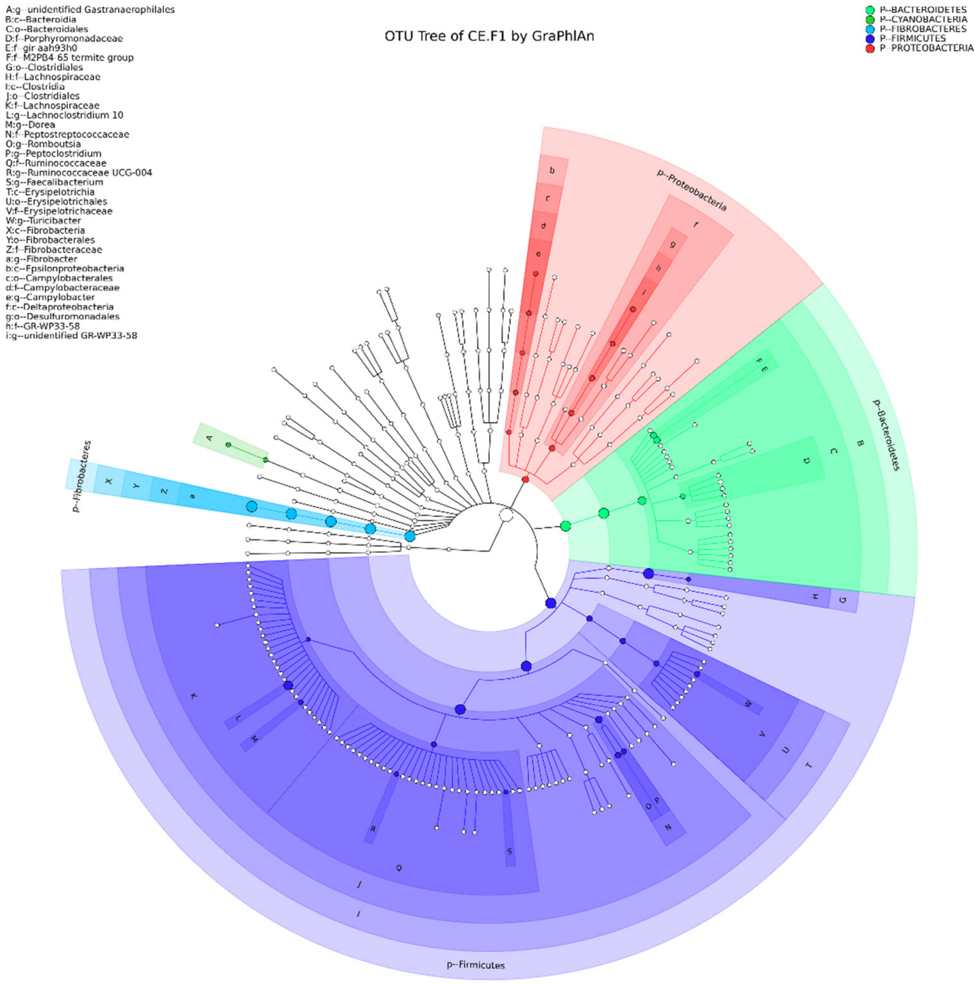

Figure S2 | Hierarchical tree of the taxa composition at each taxonomic level was constructed by GraPhlAn.

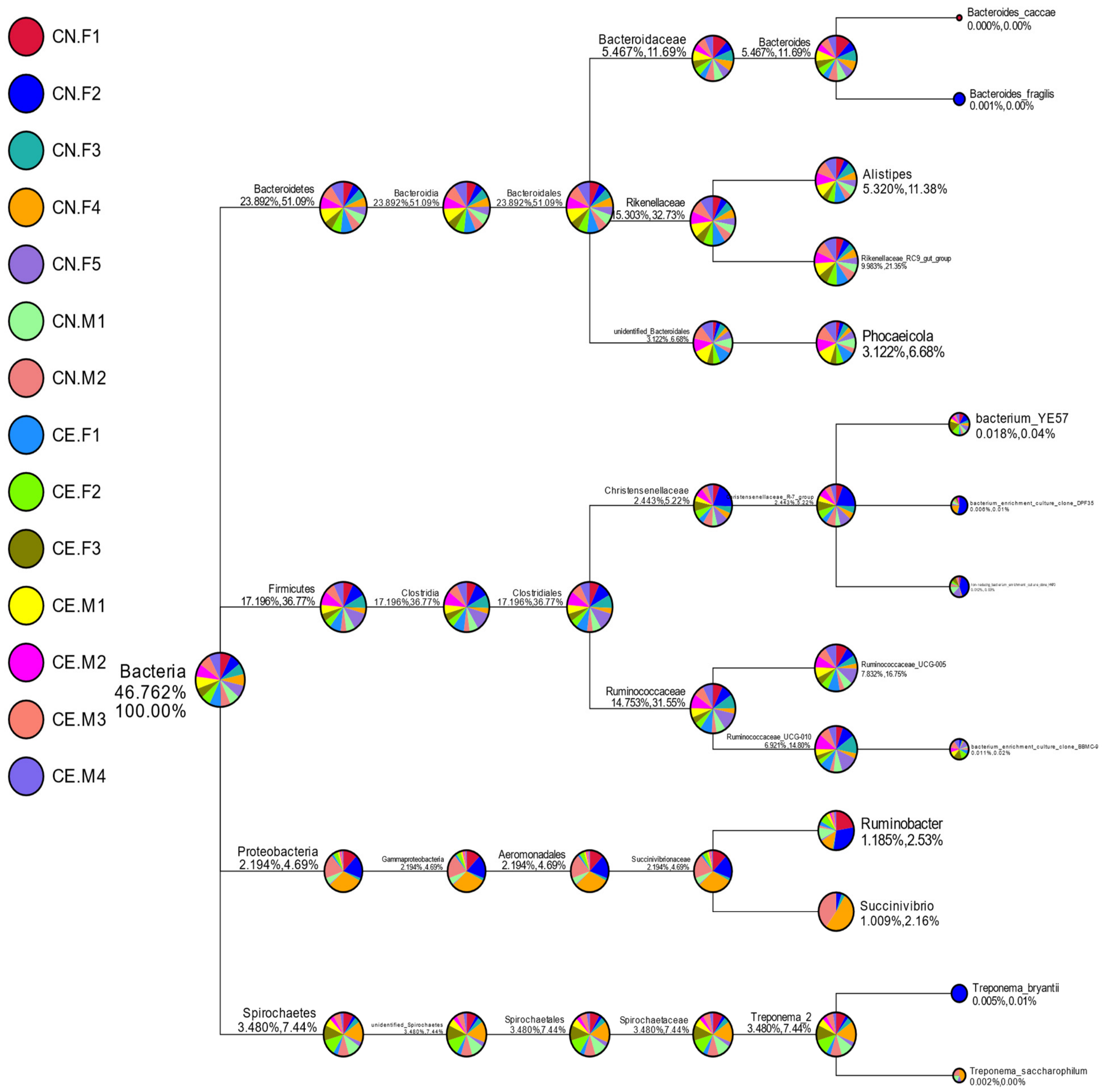

Figure S3 | The species classification tree statistics.

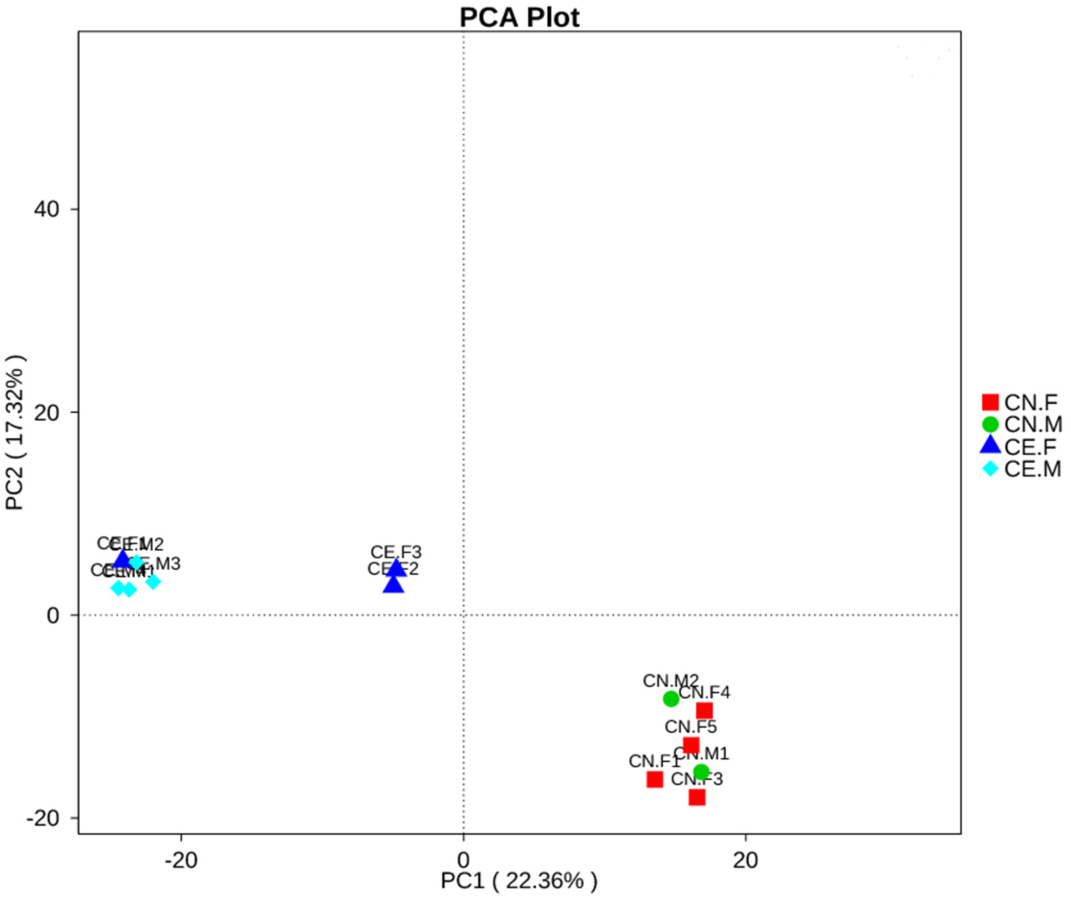

Figure S4 | Principal component analysis (PCA) plot.

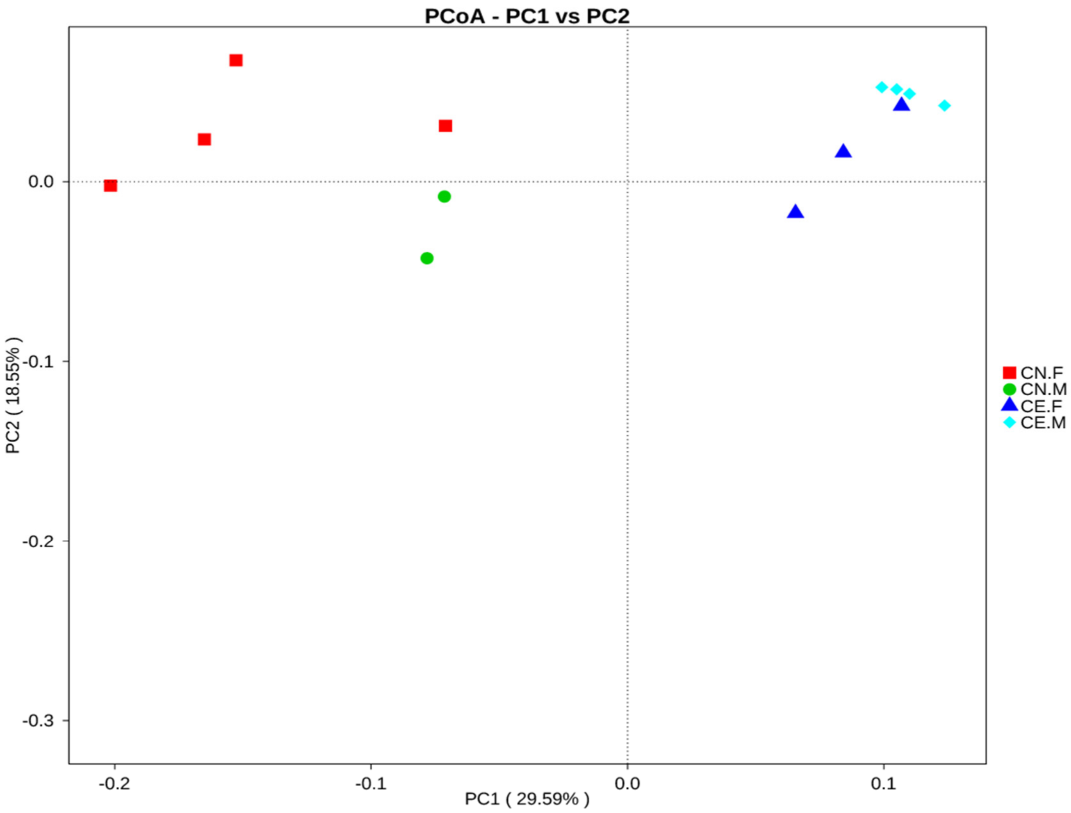

Figure S5 | Unweighted UniFrac-Principal coordinate analysis (PCoA).

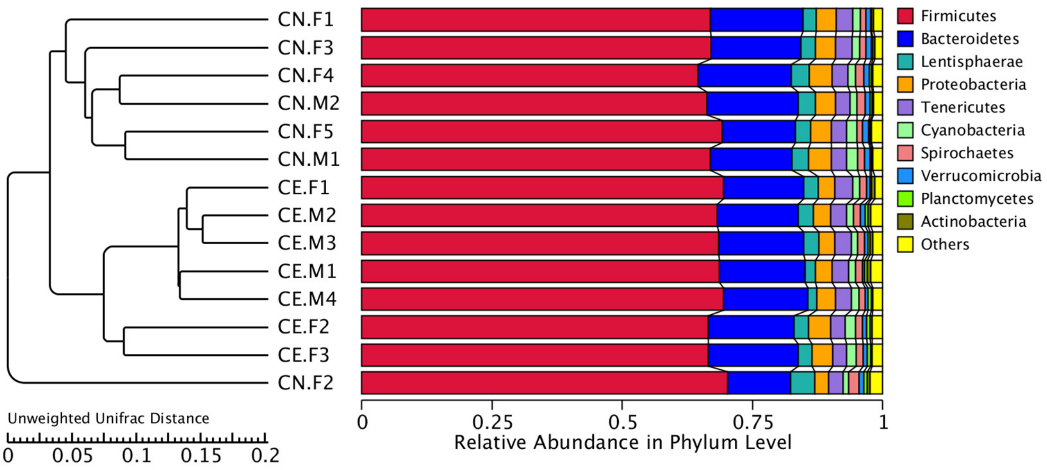

Figure S6 | Dendrograms were constructed using unweighted pair-group method using arithmetic mean (UPGMA).

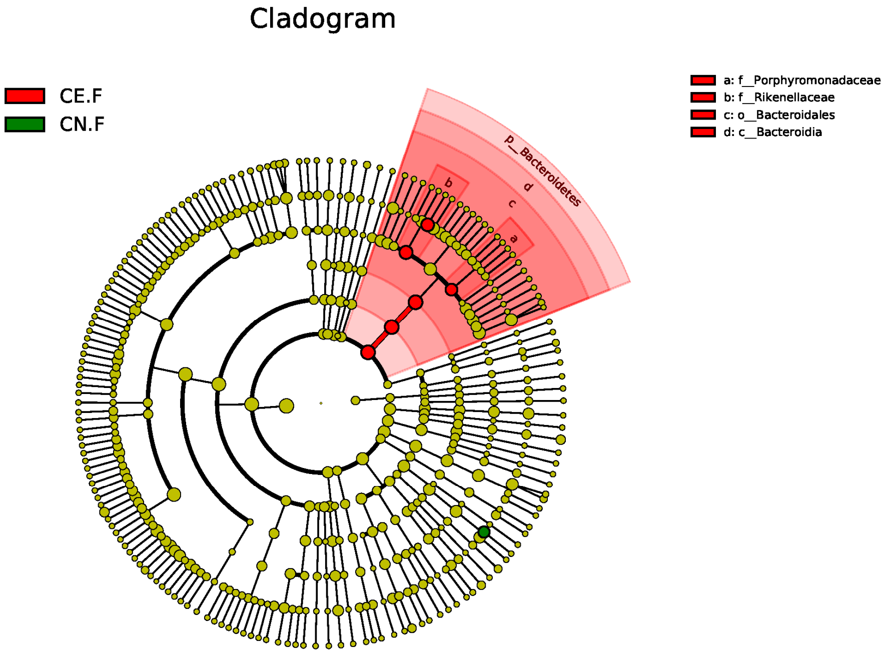

Figure S7 | Cladograms indicating the polygenetic distribution of bacterial lineages between CE.F and CN.F by linear discriminant analysis (LDA) effect size (LEfSe). Coloring principles: Species with no significant difference were uniformly colored yellow, the species of Biomarker were colored following the different group, the red node was the bacteria group that played an important role in the red group, and the green node was indicated to play an important role in the green group bacterial taxa.
